# Reflection-Aware Generation and Identification of Square Marker Dictionaries

**DOI:** 10.3390/s22218548

**Published:** 2022-11-06

**Authors:** Sergio Garrido-Jurado, Juan Garrido, David Jurado-Rodríguez, Francisco Vázquez, Rafael Muñoz-Salinas

**Affiliations:** 1 Seabery R&D, Aldebarán Building, Córdoba Science and Technology Park, 14014 Córdoba, Spain; 2 Department of Electrical Engineering and Automation, Rabanales Campus, University of Córdoba, 14071 Córdoba, Spain; 3 Department of Computer Science and Numerical Analysis, Rabanales Campus, University of Córdoba, 14071 Córdoba, Spain

**Keywords:** square markers, fiducial markers, camera localization, object tracking, augmented reality

## Abstract

Square markers are a widespread tool to find correspondences for camera localization because of their robustness, accuracy, and detection speed. Their identification is usually based on a binary encoding that accounts for the different rotations of the marker; however, most systems do not consider the possibility of observing reflected markers. This case is possible in environments containing mirrors or reflective surfaces, and its lack of consideration is a source of detection errors, which is contrary to the robustness expected from square markers. This is the first work in the literature that focuses on reflection-aware square marker dictionaries. We present the derivation of the inter-marker distance of a reflection-aware dictionary and propose new algorithms for generating and identifying such dictionaries. Additionally, part of the proposed method can be used to optimize preexisting dictionaries to take reflection into account. The experimentation carried out demonstrates how our proposal greatly outperforms the most popular predefined dictionaries in terms of inter-marker distance and how the optimization process significantly improves them.

## 1. Introduction 

Camera localization is a fundamental process in many computer vision applications, such as augmented reality [1,2], autonomous driving [3,4], or robotics [5,6]. This problem is usually tackled by finding correspondences between the real environment and their projections on the camera image, followed by a Perspective-n-Point (PnP) optimization [7] to estimate the 3D rotation and translation of the camera. The correspondences search can be performed using natural features, such as keypoints or textures [8,9,10], or using artificial markers that facilitate their detection and guarantee higher robustness, such as retroreflective spheres, VLC transmitters, LEDs, or planar markers [11,12,13,14].

**Figure 1:**
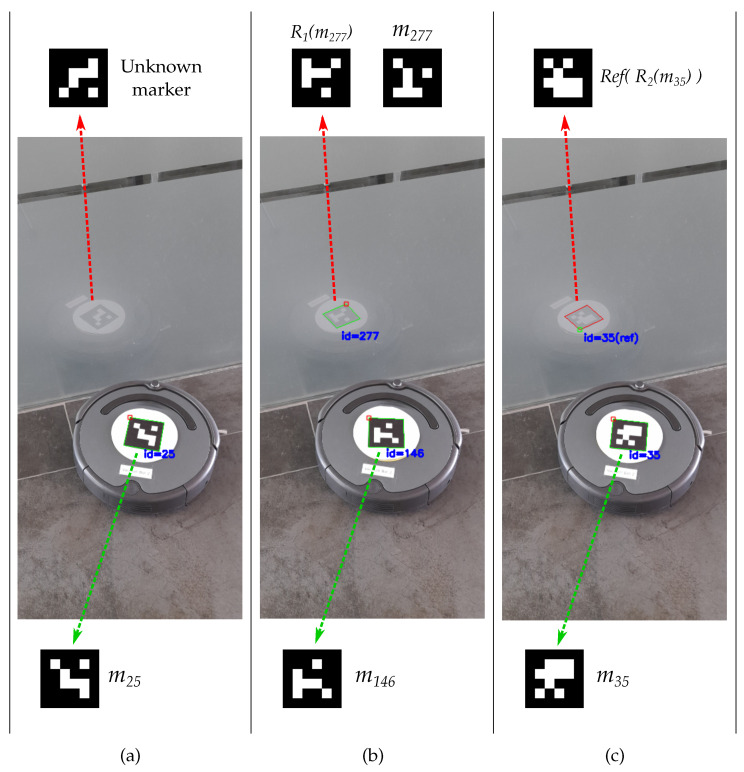
Examples of marker detection when observed through a reflective surface in a robot tracking system. In (**a**,**b**), a non-reflection-aware dictionary is used, in particular ArUco OpenCV 4 × 4 (see Table 1). Panel (**a**) shows a false negative since the reflected marker code (top) is not recognized within the dictionary. In (**b**) an inter-marker confusion error occurs because the reflected code of marker $m_{146}$ is identical to the code of marker $m_{277}$ after a rotation. Panel (**c**) employs one of the reflection-aware dictionaries generated by our proposal and the reflection-aware identification process. Thanks to that, it is possible to detect the marker even when it is reflected and also determine the kind of detection, i.e., direct or reflected.

Among the different types of flat markers, square ones are among the most widely used [15,16,17]. Square markers are composed of a black outline, which simplifies their detection in the image, and a central area for identification, usually a binary grid code that allows the application of error detection and correction techniques (see Figure 1).

Marker detection can be complex and error-prone due to different causes, such as inadequate illumination, occlusions, motion blur, or reflected observations. Some of these scenarios have been addressed in previous contributions [18,19]; however, the detection of reflected markers, i.e., seen through a mirror or reflective surface, has not received adequate attention despite being a potential source of errors. This work focuses on the detection of binary square markers in the presence of reflections.

Detection of square markers involves two steps [20,21]. The first one looks for candidates in the image, i.e., square shapes that resemble a marker, while the second one checks whether candidates are markers or something else. To perform this identification, it is necessary to verify whether the code is part of the set of valid codes in the system. This set is known as the dictionary.

A key characteristic of a dictionary is its inter-marker distance [22], which is equivalent to the minimum Hamming distance between all its markers. This value is directly related to the error correction capability and, therefore, to the false negative rate and to the inter-marker confusion rate, i.e., rate of confusing one marker into another.

The literature contains several proposals for square marker detection, usually including a public implementation. Almost all of these systems provide predefined dictionaries that cover most of the common situations. However, in some applications the predefined dictionaries are not appropriate and it is necessary to generate a custom one. Some reasons for the use of custom dictionaries include requiring a higher number of markers to cover a large area [23], using a minimum dictionary size to maximize the inter-marker distance [24], using an unusual number of bits [25], or requiring custom binary patterns, for example, to maximize the number of bit transitions [26]. Therefore, some of the available systems also propose methods to generate custom dictionaries.

**Table 1:** Summary of the public dictionaries evaluated in the experimentation.

Name	Library	Generation Method	Marker Size (n×n)	Dictionary Size (*p*)	Reflection-Aware
ARTag	ARTag	Fixed	6×6	1023	Yes
ARToolKitPlus Simple	ARToolKitPlus	Fixed	6×6	512	No
ARToolKitPlus BCH	ARToolKitPlus	Fixed	6×6	4096	No
AprilTag 16h5	AprilTag	[20]	4×4	30	No
AprilTag 25h7	AprilTag	[20]	5×5	242	No
AprilTag 25h9	AprilTag	[20]	5×5	35	No
AprilTag 36h10	AprilTag	[20]	6×6	2320	No
AprilTag 36h11	AprilTag	[20]	6×6	587	No
ArUco Original	ArUco	Fixed	5×5	1024	No
ArUco OpenCV 4×4	ArUco (OpenCV)	[21]	4×4	1000	No
ArUco OpenCV 5×5	ArUco (OpenCV)	[21]	5×5	1000	No
ArUco OpenCV 6×6	ArUco (OpenCV)	[21]	6×6	1000	No
ArUco OpenCV 7×7	ArUco (OpenCV)	[21]	7×7	1000	No
ArUco MIP 16h3	ArUco	[22]	4×4	250	No
ArUco MIP 25h7	ArUco	[22]	5×5	100	No
ArUco MIP 36h12	ArUco	[22]	6×6	250	No

ARTag [27] is one of the first libraries to propose binary-coded markers and the only one, to our knowledge, whose dictionary contemplates marker reflection, although it does not elaborate on this aspect. ARTag’s dictionary uses a predefined CRC code [28] to encode the marker identifiers and does not allow the use of other custom dictionaries, i.e., with a user-defined number of markers or number of bits. A feature introduced by ARTag, which has been adopted in some later proposals, is to provide the dictionary in a specific order so that a smaller number of markers can be picked to maximize the inter-marker distance.

ARToolKitPlus library [29] is based on its predecessor, the well-known ARToolKit [15], but introduces some improvements, including the usage of binary encoding. In its early versions, it included a predefined dictionary, known as Simple, based on repeating a 9-bit code four times. In later versions, it provides a new, more robust dictionary that uses a BCH encoding [30].

Most modern libraries, besides providing some pregenerated dictionaries, also propose dictionary generation algorithms based on some heuristics with the objective of maximizing the inter-marker distance. AprilTag [20] proposes an iterative method in which each new marker is generated by searching for a code that satisfies a minimum inter-marker distance as well as a minimum geometric complexity to increase the number of bit transitions. One of the main drawbacks is that the generation time is significantly long. AprilTag’s library contains several predefined dictionaries generated with this method.

The first versions of the ArUco library [31] included a predefined dictionary whose encoding was based on using five words of 5 bits, with 2 bits being for information and 3 bits being for redundancy. Its main drawback was that it did not account for marker rotation, resulting in a low inter-marker distance. In spite of this, this dictionary has been maintained as its use is still widespread.

In [21], ArUco authors propose a new heuristic method for generating customized dictionaries that considers the inter-marker distance. This method consists of an iterative process in which each new marker is generated based on a probability distribution that tries to maximize the inter-marker distance and the number of bit transitions. Its main disadvantage is that generation times and memory requirements are too high. The predefined dictionaries of the ArUco fork included in OpenCV [32] were generated using this method.

Finally, in [22], two methods are proposed to generate customized dictionaries using mixed integer programming (MIP) [33]. The former is the first proposal in the literature that guarantees optimal dictionaries in terms of inter-marker distance; however, it suffers from high time and space complexity, making it impractical for generating dictionaries with marker sizes larger than 3 × 3 bits. The second proposal is a suboptimal method in which a new marker is generated in each iteration, maximizing the inter-marker distance relative to the previously generated markers. This proposal achieves the best inter-marker distances to date considering only rotation, although the generation times are high for large marker sizes. The main ArUco library [31] includes several predefined dictionaries generated with this method.

**Figure 2:**
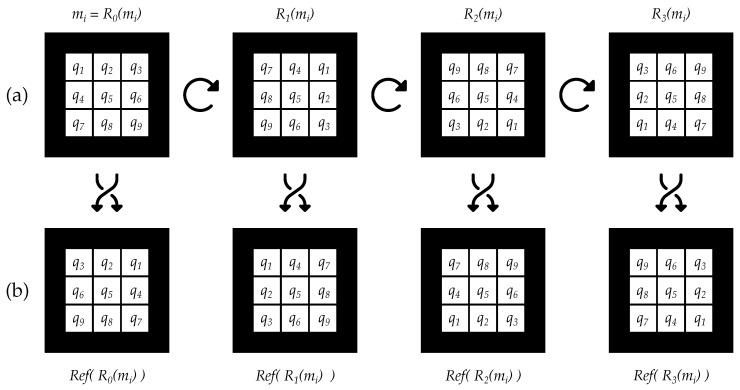
Bit permutations when applying the rotation and reflection transformations on a marker $m_{i}$ of 3×3 bits. (**a**) Original marker $m_{i}$ and its other three rotations after rotating 90, 180, and 270 degrees, respectively. (**b**) Resulting markers after applying reflection to each of the markers in (**a**). Note that reflection is applied with respect to the vertical axis but it could be performed with respect to the vertical or horizontal axis indifferently since it is applied to all 4 rotations and the result would be equivalent. The complete set of 8 markers corresponds to $A(m_{i})$, the first row corresponds to $A_{rot}{\left(m\right)}_{i}$, the second row corresponds to $A_{ref}\left(m_{i}\right)$, and the set of all markers except the original one, $m_{i}$, represents $A_{s}\left(m_{i}\right)$ (see Equations (4) and (7)).

Most of the above methods and dictionaries attempt to maximize the inter-marker distance, which is an NP-complete problem [34]. This complexity is partly because a marker can be observed in any of its four rotations (see Figure 2a) and those rotations must be considered when calculating the marker distances.

Besides being rotated, a marker could also be reflected, which would modify the disposition of its internal code, as shown in Figure 2b. This situation occurs when a marker is seen through a mirror or reflective surface, as in Figure 1, creating a source of potential errors in the pose estimation if neglected. Although previous contributions have overlooked this possibility, it can arise depending on the type of environment and the control we have over it, i.e., absence of reflective surfaces cannot always be guaranteed. Considering that artificial markers are chosen mainly for their robustness, the reflected marker scenario should also be addressed to avoid errors.

None of the previous systems, except ARTag’s predefined dictionary, contemplate the possibility of marker reflection. Because of this, in this work, we present a new method for generating and detecting reflection-aware dictionaries, with this being the first work in the literature that delves into this topic.

The rest of this paper is structured as follows. Section 2 presents a mathematical formalization of the problem. Section 3 details the proposed generation and identification methods for reflection-aware dictionaries. Finally, Section 4 presents the experimentation carried out, and Section 5 draws some conclusions.

## 2. Problem Formulation

A dictionary is the set of valid markers that can be detected in an application. In the case of binary markers, the dictionaries vary depending on the number of bits and the encoding of each marker.

An advantage of using binary markers is that error detection and correction techniques can be applied to avoid false negatives. However, if the encoding of two or more markers in the dictionary is too similar, the system could become confused in the correction and misidentify one marker as another; this is known as inter-marker confusion error [27]. To avoid this, during dictionary generation we try to maximize the inter-marker distance, which is the minimum distance within a dictionary and is directly related to the maximum number of bits that can be corrected during the identification. In the following, we derive the calculation of the inter-marker distance using a similar approach to that of [21] but adding the condition of reflected markers.

First, we define a marker, $m_{i}$, of size n×n as a vector of bits (see Figure 2):

(1) $$m_{i}=(q_{1},q_{2},q_{3}\ldots ,q_{n\times n})\;|\;q_{k}\in \left\{0,1\right\}.$$

where *i* is the marker identifier. Note that to simplify the notation we treat a marker as a binary vector rather than as a matrix.

We define D as the set of all possible binary markers with size n×n. A dictionary of markers, D, consisting of *p* markers is an element of the set $D^{p}$:

(2) $$D=\left(m_{1},m_{2},m_{3}\ldots ,m_{p}\right)\in D^{p}.$$

The goal, therefore, is to find a dictionary, D, that maximizes its inter-marker distance, τ(D):

(3) $$D^{*}=\underset{D\in D^{p}}{\mathrm{argmax}}\left\{\tau (D)\right\}.$$

Note that, when detecting a marker in an image, a marker may be rotated with respect to its original position and, hence, the extracted bits will also be rotated (see Figure 2a). Similarly, when a marker is reflected, the bits are also mirrored (see Figure 2b). When comparing the distance between two markers, we must consider all these possible transformations. We define the analogous set of a marker, $A(m_{i})$, as the set of markers obtained by applying the rotation and reflection transformations:

(4) $$\begin{array}{cc} \; & A\left(m_{i}\right)=A_{rot}\left(m_{i}\right)\cup A_{ref}\left(m_{i}\right),\\ \; & A_{rot}\left(m_{i}\right)=\overset{3}{\underset{l=0}{⋃}}R_{l}\left(m_{i}\right),\\ \; & A_{ref}\left(m_{i}\right)=\overset{3}{\underset{l=0}{⋃}}Ref\left(R_{l}\left(m_{i}\right)\right), \end{array}$$

where $A_{rot}\left(m_{i}\right)$ and $A_{ref}\left(m_{i}\right)$ correspond to the marker sets obtained from rotation and reflection transformations, respectively, $R_{l}$ applies a rotation of l×90 degrees clockwise to a marker, and Ref reflects a marker. Both transformations consist of a permutation in the bit positions of the marker, as shown in Figure 2. A marker is considered equivalent to all markers in its analogous set, since it can be observed in any of its forms.

Hence, the distance between two markers considering all possible transformation is the minimum Hamming distance between any markers in each other’s analogous sets:

(5) $$d\left(m_{i},m_{j}\right)=\min_{m_{k}\in A\left(m_{j}\right)}\left\{\;ham\left(m_{i},m_{k}\right)\;\right\},$$

ham() being the function that computes the Hamming distance between two markers.

Besides distinguishing between different markers, we often want to identify each of the marker corners unambiguously in order to perform a correct pose estimation. In other words, it is necessary to identify the specific rotation or reflection at which a marker has been detected. To achieve this, we must consider the distance between a marker and the rest of the elements of its own analogous set. We call this measure the marker self-distance:

(6) $$d_{s}\left(m_{i}\right)=\min_{m_{k}\in A_{s}\left(m_{i}\right)}\left\{\;ham\left(m_{i},m_{k}\right)\;\right\},$$

where $A_{s}\left(m_{i}\right)$ is the analogous set of $m_{i}$ without considering the marker itself:

(7) $$A_{s}\left(m_{i}\right)=A\left(m_{i}\right)-\left\{m_{i}\right\}$$

Finally, we can define the inter-marker distance of a dictionary, τ(D), as the minimum distance between all self-distances and distances between pairs of markers in the dictionary:

(8) $$\tau \left(D\right)=\min\left\{\min_{\begin{array}{c} m_{i},m_{j}\in D\\ m_{i}\neq m_{j} \end{array}}\left\{d(m_{i},m_{j})\right\},\min_{m_{i}\in D}\left\{d_{s}\left(m_{i}\right)\right\},\right\}.$$

As defined in Equation (3), this is the measure we want to maximize when generating a dictionary since it is directly related to the error correction capability. The longer the distance, the further apart the markers are and the lower the probability of confusing one marker with another one when correcting bits. Specifically, the maximum number of bits that can be corrected in a dictionary without the danger of causing an inter-marker confusion error is $\left\lfloor \left(\tau \left(D\right)-1\right)/2\right\rfloor$.

## 3. Proposed Solution

This section presents the proposed method for generating reflection-aware dictionaries. The method is divided into two parts. In the first part, an initial dictionary is generated (Section 3.1), while in the second part, the previous dictionary is optimized by selecting a subset of markers to maximize the inter-marker distance (Section 3.2). Finally, we present the reflection-aware identification process for detecting markers in an image (Section 3.3).

### 3.1. Initial Dictionary Generation

Our proposal starts generating an initial dictionary whose marker codes are as far from each other as possible. The generation of optimal dictionaries is an NP-complete problem [22]; hence, a heuristic approach is necessary. We propose an iterative algorithm that adds a new marker at each iteration until the desired dictionary size is reached.

In each iteration, the new marker is compared with the dictionary generated up to that moment. Therefore, it is necessary to define some concepts related to the distance between a marker and a dictionary. We call $H(m_{i},D)$ to the multiset composed by all Hamming distances of a marker $m_{i}$ with respect to all markers in a dictionary D:

(9) $$H\left(m_{i},D\right)=\underset{\begin{array}{c} m_{j}\in D\\ m_{k}\in A\left(m_{j}\right) \end{array}}{⋃}\;ham\left(m_{i},m_{k}\right).$$

We further define $d_{H}\left(m_{i},D\right)$ as the minimum distance within $H(m_{i},D)$ and $f_{H}\left(m_{i},D\right)$ to the multiplicity of this minimum distance, i.e., the number of times it is repeated in the multiset:

(10) $$\begin{array}{cc} \; & d_{H}\left(m_{i},D\right)=min\left(H\left(m_{i},D\right)\right),\\ \; & f_{H}\left(m_{i},D\right)=\sum_{\begin{array}{c} h\in H(m_{i},D)\\ h=d_{H}\left(m_{i},D\right) \end{array}}\;1. \end{array}$$

At each iteration *t*, a new marker, $m_{t}$, is randomly initialized by assigning a 0 or a 1 to each bit $q_{k}$ with the same probability:

(11) $$\left.\begin{array}{c} P(q_{k}=0)=0.5\\ P(q_{k}=1)=0.5 \end{array}\right\}\forall q_{k}\in m_{t}.$$

Then, a greedy algorithm is used to increase the distances of $m_{t}$ to the current dictionary, $D_{t}$. A greedy algorithm consists of taking small steps that improve the result until no step is found that produces a better solution. In our approach, each of these steps corresponds to a modification of a single bit of $m_{t}$. We call $m_{t}^{b}$ to the resulting marker after modifying the bit in position *b* of $m_{t}$:

(12) $$q_{k}=\left\{\begin{array}{c} \neg r_{k}\;\;\;if\;k=b\\ r_{k}\;\;\;otherwise \end{array}\right.\;\;\;\forall q_{k}\in m_{t}^{b}\;\;and\;\;\forall r_{k}\in m_{t}$$

where $q_{k}$ represents the bits in $m_{t}^{b}$ and $r_{k}$ the bits in $m_{t}$. Among all the bits that can be modified at each step, we choose the one that maximizes the sum of distances in $H(m_{t},D_{t})$:

(13) $$b^{*}=\underset{b\in n\times n}{\mathrm{argmax}}\left\{\sum_{h\in H(m_{t}^{b},D_{t})}\;h\;\;\;\;\right\},$$

provided that, after the bit modification, the marker meets the following constraints that guarantee a minimum quality:

(14) $$d_{s}\left(m_{t}^{b}\right)\geq d_{H}\left(m_{t}^{b},D_{t}\right),$$

(15) $$d_{H}\left(m_{t}^{b},D_{t}\right)\geq d_{H}\left(m_{t},D_{t}\right),$$

(16) $$d_{H}\left(m_{t}^{b},D_{t}\right)>d_{H}\left(m_{t},D_{t}\right)\;\vee \;f_{H}\left(m_{t}^{b},D_{t}\right)<f_{H}\left(m_{t},D_{t}\right).$$

The first condition guarantees that the self-distance of the new marker is not less than the distance to any other marker in the dictionary. The second condition ensures that the minimum distance to the dictionary does not decrease after the bit change. Finally, the third condition guarantees that, if the minimum distance has not increased, at least the number of its occurrences has decreased.

The bit modification process is repeated until there are no modifications that meet the above constraints. In that case, the marker cannot be improved any further, so it is added to the dictionary and the next iteration, t+1, is started. The dictionary generation process continues until the desired number of markers has been reached. The proposed method pseudocode is shown in Algorithm 1.
**Algorithm 1** Initial dictionary generation.**Input:** int *n*,   int *p*▹ Marker size and dictionary size**Output:** Dictionary $D_{out}$1: $D_{1}\leftarrow \emptyset$2: **for** t←1 to *p* **do**3:    $m_{t}\leftarrow$ generate_random_marker(*n*)▹ See Equation (11)4:    bool improvement_found ← false5:    **do**6:        improvement_found ← false7:        int best_b ←08:        int best_sum_hamming ←09:        **for** b←1 to n×n **do**10:           $m_{t}^{b}\leftarrow$ apply_bit_change($m_{t},b$)▹ See Equation (12)11:           **if** $d_{s}\left(m_{t}^{b}\right)<d_{H}\left(m_{t}^{b},D_{t}\right)$ **then continue**▹ See Equation (14)12:           **if** $d_{H}\left(m_{t}^{b},D_{t}\right)<d_{H}\left(m_{t},D_{t}\right)$ **then continue**▹ See Equation (15)13:           **if** $d_{H}\left(m_{t}^{b},D_{t}\right)=d_{H}\left(m_{t},D_{t}\right)$ **and**                    $f_{H}\left(m_{t}^{b},D_{t}\right)\geq f_{H}\left(m_{t},D_{t}\right)$ **then continue**▹ See Equation (16)14:           int sum_hamming ←0;15:           **for each** $h\in H(m_{t}^{b},D_{t})$**do**▹ See Equation (9)16:               sum_hamming ← sum_hamming + *h*17:           **end for**18:           **if** sum_hamming > best_sum_hamming **then**19:               best_sum_hamming ← sum_hamming20:               best_b ←*b*21:               improvement_found ← true22:           **end if**23:        **end for**24:        **if** improvement_found **then** $m_{t}\leftarrow m_{t}^{best\_b}$25:    **while** improvement_found26:    $D_{t+1}\leftarrow D_{t}⋃m_{t}$27: **end for**


### 3.2. Dictionary Optimization

Although Algorithm 1 allows to generate a dictionary maximizing the distances between markers, it is not optimal and can produce low-quality markers that penalize the inter-marker distance. It is therefore advisable to generate more markers than required and apply a second step to select a subset of markers that maximize the inter-marker distance. This section explains the proposed method for marker selection.

The algorithm consists of an iterative process in which, in each iteration, we look for the optimal subset of markers for a specific value of inter-marker distance.

It starts with a large value of inter-marker distance, which is relaxed, i.e., decremented, on each iteration. In this manner, we begin by looking for small sets with large inter-marker distance, and as we progress we seek larger sets with smaller inter-marker distance. The process finishes when a subset with sufficient markers has been found or when all the markers in the dictionary have been selected. For the initial inter-marker distance, we use the largest self-distance of any marker in the dictionary since, by definition, it is always greater than or equal to the maximum theoretical inter-marker distance (see Equation (8)).

More precisely, for each iteration we have to solve the problem of, given an input dictionary D, obtaining the largest subset of markers, $D_{u}\subseteq D$, such that $\tau (D_{u})\geq u$, where *u* is the target inter-marker distance for that iteration.

To solve this problem we represent the dictionary as an undirected graph, $G_{u}=\left(V_{u},E_{u}\right)$. The graph will contain a vertex $v_{i}\in V_{u}$ for each marker $m_{i}\in D$ whose self-distance, $d_{s}\left(m_{i}\right)$, is greater than or equal to *u*. Furthermore, two vertices, $v_{i}$ and $v_{j}$, will be connected by an edge, $e_{i,j}\in E_{u}$, if the distance between their corresponding markers, $m_{i}$ and $m_{j}$, is greater than or equal to *u*:

(17) $$\begin{array}{cc} \; & V_{u}=\left\{v_{i}\;:\;m_{i}\in D\;\wedge \;d_{s}\left(m_{i}\right)\geq u\right\},\\ \; & E_{u}=\left\{e_{i,j}\;:\;m_{i},m_{j}\in D\;\wedge \;d\left(m_{i},m_{j}\right)\geq u\right\}. \end{array}$$

The solution to our problem is obtained by finding the maximum clique of the graph. A clique is a subset of vertices that are all connected to each other, which in our problem means that the distances between all markers in the clique are greater than or equal to *u*; in other words, $\tau (D_{u})\geq u$. The maximum clique is the largest clique within a graph and thus the largest subset of markers with inter-marker distance greater than or equal to *u*.

Estimating the maximum clique is a classical problem in graph theory and there are numerous algorithms to solve it [35,36,37]. In our case, we have opted for the approximate coloring algorithm proposed in [38] due to its availability, simplicity, and efficiency.

The pseudocode of the dictionary optimization is shown in Algorithm 2.
**Algorithm 2** Dictionary optimization.**Input:** Dictionary $D_{in}$,   int target_dictionary_size**Output:** Dictionary $D_{out}$▹ Optimized dictionary1: $u\leftarrow \max\left\{{⋃}_{m_{i}\in D_{in}}d_{s}\left(m_{i}\right)\right\}$▹ Maximum self-distance2: **while** u≥0
 **do**3:    $G_{u}\leftarrow$ generate_graph($D_{in}$, *u*)4:    $D_{u}\leftarrow$ find_maximum_clique($G_{u}$)5:    **if** $|D_{u}|\geq$ target_dictionary_size **then**6:        $D_{out}\leftarrow D_{u}$7:        **return**8:    **end if**9:    u←u−110: **end while**


Additionally, the optimization algorithm can also be applied to any predefined dictionary from public libraries. In this manner, they can be optimized by taking the subset of markers that maximizes the inter-marker distance when considering reflection. This is especially interesting for those applications that are already using a marker system and do not want to switch to a new dictionary. The results of these optimizations are shown in Section 4.3.

Finally, although this work focuses on marker reflection, it must be noted that the proposed algorithms, Algorithm 1 and Algorithm 2, can be easily adapted to only consider rotation and ignore reflection. This only requires removing the reflection term, $A_{ref}\left(m_{i}\right)$, from Equation (4).

### 3.3. Identification of Reflected Markers

While each marker system proposes its own pipeline for analyzing an image and detecting markers, they typically share two fundamental steps. First, the image is processed to find candidates, i.e., square shapes with a black border that resemble a marker, and then each candidate is examined to verify whether it is a marker or not. As part of this second step, it is necessary to extract the bits of the marker from the image and determine if they belong to a valid dictionary code or not.

In libraries such as ArUco or AprilTag, the extracted code, $m_{i}$, is compared with all the markers in the dictionary, D, and it is identified as a valid marker if there is any marker in the dictionary, $m_{j}$, whose distance, $d(m_{i},m_{j})$, is less than or equal to $\left\lfloor \left(\tau (D\right)-1)/2\right\rfloor$, which is the maximum number of bits that can be corrected without risking an inter-marker confusion error.

The novelty of our proposal is that it also accounts for reflection in the identification process. In particular, the algorithm finds the closest marker in the dictionary when estimating the distances $d(m_{i},m_{j})$ by considering the reflection analogous set, $A_{ref}\left(m_{j}\right)$, in Equation (4).

Furthermore, not only can we identify a marker, but we can also distinguish whether it has been detected directly or reflected. To achieve this, it is sufficient to check whether the minimum Hamming distance between $m_{i}$ and $m_{j}$ is obtained with respect to the set $A_{ref}\left(m_{j}\right)$, indicating reflected detection, or to the set $A_{rot}\left(m_{j}\right)$, indicating direct detection. This information is necessary to identify each of the marker corners uniquely, but it can also be useful for other purposes. For example, an augmented reality application could display the virtual objects inverted in accordance with the reflected marker, or a robotic navigation application could ignore the reflected markers to avoid introducing potential errors in the trajectory estimation. Algorithm 3 summarizes the reflection-aware identification process.
**Algorithm 3** Reflection-aware marker identification.**Input:** Dictionary D,   Marker Candidate $m_{i}$**Output:** bool is_marker,   Identifier marker_id,   bool is_reflected1: **for each** $m_{j}\in D$
 **do**2:    **if** $d\left(m_{i},m_{j}\right)\leq \left\lfloor \left(\tau (D\right)-1\right)/2\rfloor$ **then**▹ See Equation (5)3:        is_marker ← true4:        marker_id ←j5:        **if** $d_{H}\left(m_{i},A_{ref}\left(m_{j}\right)\right)<d_{H}\left(m_{i},A_{rot}\left(m_{j}\right)\right)$ **then**▹ See Equation (10)6:           is_reflected ← true7:        **else**8:           is_reflected ← false9:        **end if**10:        **return**11:    **end if**12: **end for**13: is_marker ← false


Figure 1 shows several cases of reflected marker detection. In the first two, Figure 1a,b, a non-reflection-aware dictionary is used, namely, the ArUco OpenCV 4 × 4 dictionary (see Table 1). In the first scenario, Figure 1a, a false negative is produced as the detection process does not consider reflection and the code of the reflected marker does not correspond to any marker in the dictionary. The second case, Figure 1b, is more critical as the code of the original marker ($m_{146}$), when reflected, is identical to that of another marker in the dictionary after being rotated ($m_{277}$), confusing the system and causing an inter-marker confusion error.

On the other hand, in Figure 1c, the same situation is presented with a dictionary generated by our proposal, which does consider reflection, and the identification process of Algorithm 3. In this case, the marker is correctly identified and, in addition, it is possible to distinguish whether it is reflected or not.

## 4. Experimentation and Results

**Figure 3:**
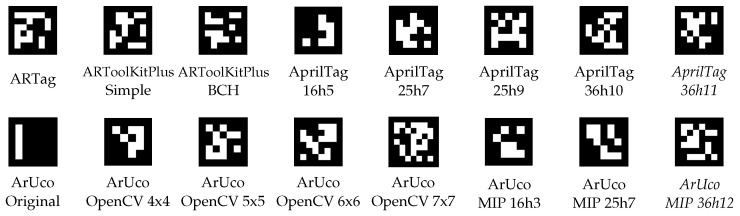
First marker of each of the dictionaries listed in Table 1.

This section details the experimentation carried out to validate our proposed dictionary generation compared to the public dictionaries of the most popular libraries for marker detection. Table 1 lists the dictionaries of other libraries against which we compared and some of their most relevant features. Note that the ArUco OpenCV dictionaries refer to those available in the OpenCV fork of ArUco [32] while ArUco MIP dictionaries refer to those available in the main ArUco library [31]. ArUco Original refers to the first ArUco dictionary, which is included in both implementations. Figure 3 shows the first marker of each of the dictionaries listed in Table 1.

Section 4.1 compares the inter-marker distance of the dictionaries generated by our proposal with those in Table 1. In Section 4.2, we study the generation times of our proposal. Finally, in Section 4.3, the dictionary optimization algorithm is applied to the dictionaries in Table 1 and the results are presented.

### 4.1. Inter-Marker Distance

This section analyzes the inter-marker distance of the dictionaries generated by our proposal and the public dictionaries listed in Table 1.

To evaluate our proposal, we generated dictionaries with markers of the most common sizes, namely 4 × 4, 5 × 5, and 6 × 6 bits. Regarding the dictionary size, we consider that 250 markers are enough to cover most of the use cases and to compare with the rest of the dictionaries in the literature. In our experience, it is sufficient to generate six to eight times the desired number of markers with Algorithm 1. Therefore, for each size, a total of 2000 (250×8) markers were generated using Algorithm 1, and a subset of 250 markers was selected using Algorithm 2. For each size, 30 different dictionaries were generated and the results were averaged. We chose 30 samples, as this is the minimum number to assure a normal distribution according to the central limit theorem [39]. To limit the search time of the maximum clique algorithm, a timeout of 150 s was configured. After the timeout, the largest clique found up to that moment is returned.

The inter-marker distance was estimated for each dictionary size from 1 to 250 markers. For the public dictionaries in Table 1, subsets of markers up to size 250 were taken in increasing order. This is appropriate since some dictionaries provide a specific order that maximizes their inter-marker distance.

**Figure 4:**
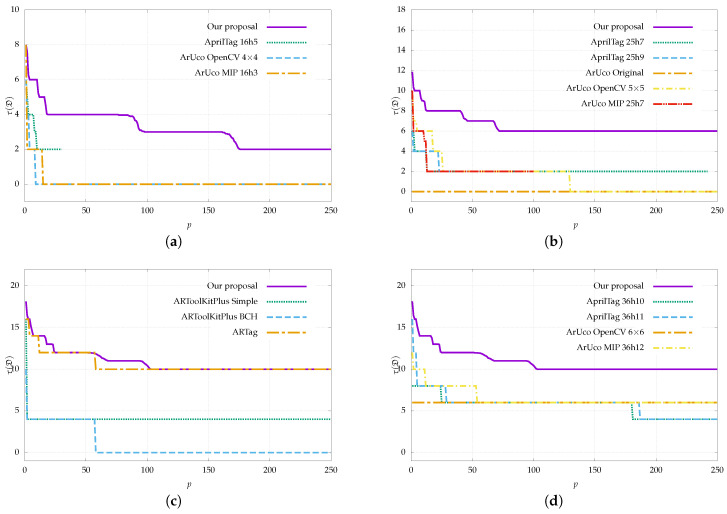
Inter-marker distance as a function of dictionary size for the proposed generation method compared to public dictionaries (see Table 1). The results are shown considering reflection and are broken down for different marker sizes. The 6 × 6 bit dictionaries are divided into two plots for easier visualization. Higher values indicate a larger inter-marker distance and, therefore, a higher error correction capability. Our proposal clearly outperforms all methods, including ARTag, which is the only one that considers reflection. (**a**) 4 × 4 bits, (**b**) 5 × 5 bits, (**c**) 6 × 6 (1/2), (**d**) 6 × 6 (2/2).

The inter-marker distance was calculated considering the formulation in Section 2, which introduces the reflection term in order to evaluate the error correction capability in the presence of reflection. Figure 4 shows the results.

It can be observed that the inter-marker distance of the dictionaries generated by our approach outperforms the other dictionaries in virtually all cases. This is expected, as the rest of the dictionaries are not reflection-aware; however, it is worth noting that the advantage is highly significant since the results are doubled in most cases. For example, if we take a dictionary of 50 markers of size 5 × 5 bits, we can observe that the maximum distance achieved by the rest of the dictionaries is two; this means that erroneous bits cannot be corrected ($\lfloor \left(\tau \left(D\right)-1\right)/2\rfloor =0$) and it would only take two incorrect bits to produce an inter-marker confusion error. On the other hand, in that same case, our proposal achieves an inter-marker distance of seven, which means that it can correct up to three erroneous bits and it would need four incorrect bits for an inter-marker confusion error to occur.

The only dictionary that comes close to the results of our proposal is that of ARTag, as it is the only one that considers the possibility of reflection. Even so, our proposal outperforms ARTag in nearly all cases. Although it is difficult to appreciate, the only exception is for dictionary sizes between 52 and 57, where ARTag reaches an inter-marker distance of 12 and some executions of our proposal drop to 11. In any case, this only represents 0.0013% of the total number of executions, so we can state that our method clearly outperforms ARTag’s dictionary. Apart from ARTag and our proposal, the best performing dictionary families are AprilTag and ArUco MIP. The other ArUco dictionaries and, in particular, the ARToolKitPlus dictionaries show worse results.

It is worth noting that some dictionaries have an inter-marker distance of 0. This is especially critical as it means that one marker could be confused with another simply by being reflected, even if there is no error in the extracted bits. Some examples are the ArUco Original, ARToolKitPlus BCH, or some of the ArUco OpenCV dictionaries. An example of this error can be seen in Figure 1b.

Most of the plots have a staircase shape in which each step down represents the decrease of the inter-marker distance as the dictionary size increases. In general, these steps are larger as we reduce the inter-marker distance requirement since it is easier to find more markers that meet that requirement. In addition, these steps are more frequent in our method and in ARTag since they are the only ones that consider reflection. In the rest of the dictionaries, the decrease of the inter-marker distance is faster and less progressive since they are not designed for reflection.

Finally, regarding marker size, we observe that, as expected, the inter-marker distance increases when using larger markers. In general, a large marker size allows for larger dictionaries and larger inter-marker distances. For example, using our proposal, a dictionary of 150 markers of 4×4 bits reaches an inter-marker distance of 3 while a dictionary of the same size and markers of 6×6 bits reaches a value of 10. On the other hand, a small marker size permits markers to be more easily detected in the image since the bits occupy more pixels. In the end, the choice of marker size will depend on the specific application and aspects such as the working distance, the physical size of the marker, or the number of markers needed.

### 4.2. Generation Time

In this section, we study the dictionary generation times of our proposal and compare them with the most popular dictionary generation algorithms in the literature. In particular, we compare against the AprilTag generation method [20], the ArUco generation method based on mixed-integer programming (ArUco MIP) [22], and the ArUco iterative generation method [21], which we refer to as ArUco OpenCV since the dictionaries of the ArUco fork in OpenCV were created using this approach. Note that these methods are the ones used to generate the custom dictionaries of Table 1. It is also noteworthy that ARTag, whose dictionary is the only one that considers reflection, just provides a predefined dictionary and not a method to generate custom dictionaries.

**Figure 5:**
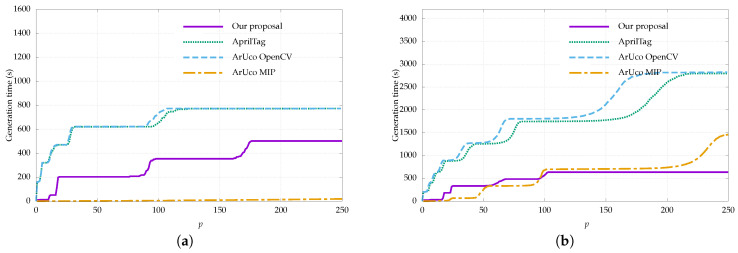
Generation times as a function of dictionary size for the proposed generation method compared to AprilTag, ArUco OpenCV, and ArUco MIP counterparts. Results are shown for marker sizes of (**a**) 4 × 4 and (**b**) 6 × 6 bits.

The generation was configured in a similar way to that proposed in [22]. All tests were performed using a system equipped with an Intel Core i7-3930K 3.20 GHz processor and 16 GB of RAM. The results of our proposal correspond to the dictionaries generated for the experimentation of Section 4.1. Figure 5 shows the generation times for two typical marker sizes of 4 × 4 and 6 × 6 bits.

The methods with the shortest generation times are our proposal and ArUco MIP. For small marker sizes, i.e., 4 × 4 bits, ArUco MIP achieves the best results with generation times of a few seconds, while our method requires more than 8 min to generate a dictionary of 250 markers. On the other hand, for larger marker sizes, namely, 6 × 6 bits, our proposal has the shortest generation times, taking about 10 min, while the second best option, ArUco MIP, takes slightly more than 24 min. The slopes in the plots where the times increase sharply correspond to those cases where the timeout has been reached. It can be seen that the steps in the plots are larger as the dictionary size increases. This is because at each slope the inter-marker distance requirement is reduced, and it is easier and faster to find new markers that meet that constraint.

In any case, the dictionary generation is normally performed offline and only once; thus, the generation times are not critical as long as they are not prohibitive.

### 4.3. Optimizing Public Dictionaries 

This section evaluates the effect of the optimization algorithm from Section 3.2 when applied to the public dictionaries in Table 1. The goal is to check whether we can reuse existing dictionaries that a priori do not consider reflection by taking a subset of markers that maximizes the inter-marker distance. This is especially interesting for those applications that intend to use a preexisting dictionary from a public library but still want to consider the possibility of reflection.

In addition to applying the optimization for reflection, we also evaluated the improvement considering only the possibility of rotation by eliminating the term $A_{ref}\left(m\right)$ from Equation (4). This analysis is interesting since almost all the predefined dictionaries were generated considering only rotation, and we can verify if our optimization process is able to improve the dictionary even in this case.

**Figure 6:**
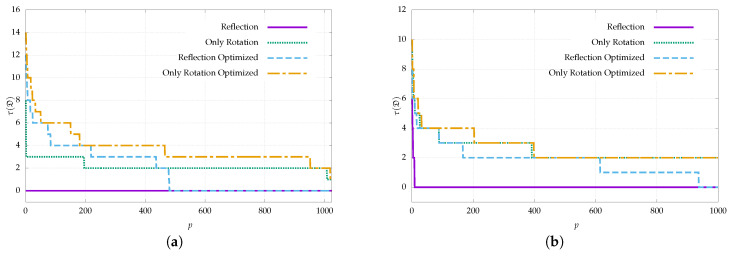

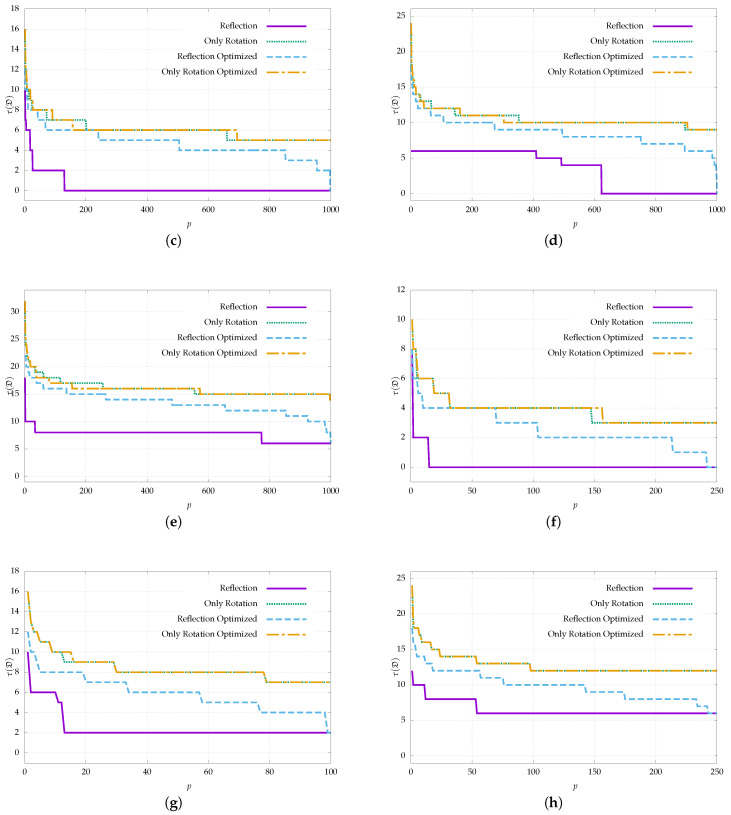
Inter-marker distance as a function of dictionary size for the ArUco family dictionaries (see Table 1) before and after applying the proposed optimization (Algorithm 2). Results are shown considering reflection as well as considering only rotation. When considering reflection, the optimized dictionaries obtain equal or better results in all cases. See Figure 7 for the rest of the dictionaries. (**a**) ArUco Original, (**b**) ArUco OpenCV 4 × 4, (**c**) ArUco OpenCV 5 × 5, (**d**) ArUco OpenCV 6 × 6, (**e**) ArUco OpenCV 7 × 7, (**f**) ArUco MIP 16h3, (**g**) ArUco MIP 25h7, (**h**) ArUco MIP 36h12.

**Figure 7:**
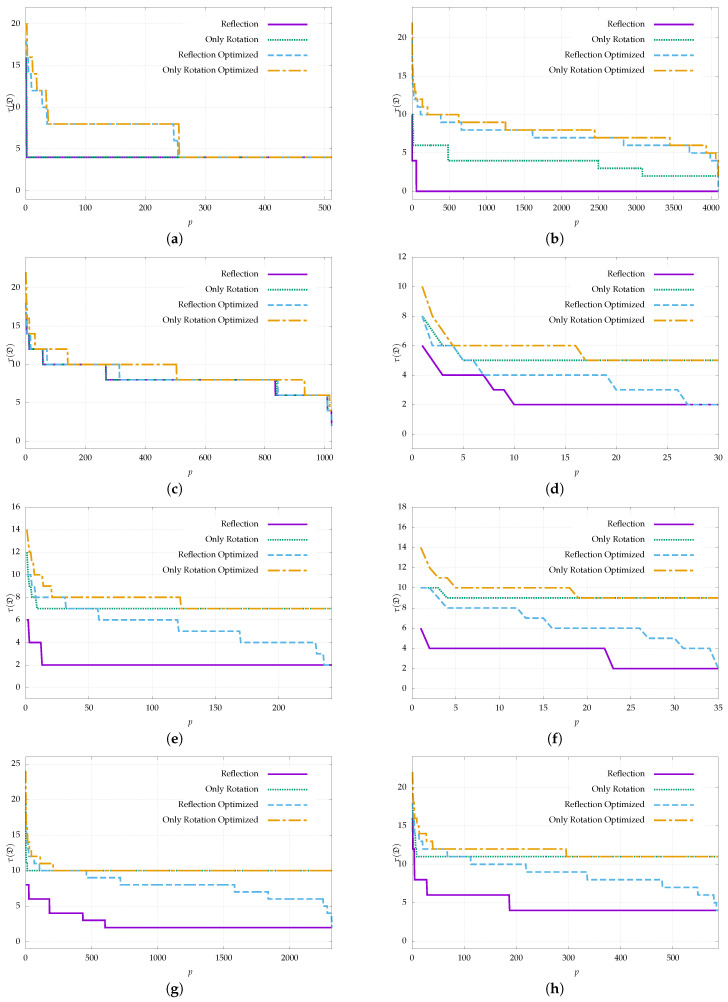
Inter-marker distance as a function of dictionary size for several of the public dictionaries in Table 1 before and after applying the proposed optimization (Algorithm 2). Results are shown considering reflection as well as considering only rotation. When considering reflection, the optimized dictionaries obtain equal or better results in all cases, even when comparing with the reflection-aware dictionary of ARTag. See Figure 6 for the rest of the dictionaries. (**a**) ARToolKit+ Simple, (**b**) ARToolKit+ BCH, (**c**) ARTag, (**d**) AprilTag 16h5, (**e**) AprilTag 25h7, (**f**) AprilTag 25h9, (**g**) AprilTag 36h10, (**h**) AprilTag 36h11.

Figure 6 and Figure 7 show the inter-marker distance results for each dictionary and as a function of the dictionary size. For each case, the results are shown before and after applying the optimization process and considering reflection or only rotation. Optimization was applied to the full size of each dictionary. Figure 6 shows the results for all the dictionaries of the ArUco family, while Figure 7 shows the same for the rest of the dictionaries in Table 1.

It can be seen that, when considering reflection, we obtain better results in nearly all cases and in the rest we equal them, but we never obtain worse results. The improvement is almost always highly significant. For instance, the ArUco OpenCV 6 × 6 dictionary of size 800 achieves an inter-marker distance of 0 before optimizing, which makes it unusable since one marker can be confused with another simply by being reflected even if the bit extraction is perfect. After applying the optimization, an inter-marker distance of seven is achieved, which allows correcting up to three incorrect bits.

The only dictionary where the improvement is not as remarkable is the case of ARTag, since it already considers reflection. Still, we achieve equal or better results in all cases. Some improvements can be observed, for example, for dictionaries sizes close to 300.

Note that some dictionaries, such as ARToolKitPlus BCH and several dictionaries of the ArUco family, achieve an inter-marker distance of zero before optimization, which makes them unusable if we want to take reflection into account. After applying the optimization, almost none of the dictionaries reach a minimum distance of zero. The only exceptions are ArUco Original, ArUco OpenCV 4 × 4, and ArUco MIP 16h3; hence, it is not recommended to use these dictionaries when the input images might contain reflections, even after applying the optimization process.

When we only take rotation into account, the optimization results also improve or equal the inter-marker distance in most cases, although the improvement is not as significant as when reflection is considered. It only obtains worse results for some dictionary sizes in ArUco OpenCV, namely, 5 × 5, 6 × 6, and 7 × 7 bits. The reason for the worsening is that the maximum clique search has reached the timeout more frequently, resulting in a suboptimal result. In several cases, the shapes of the plots are similar before and after optimization. This occurs because those dictionaries are close to the optimum and have little room for improvement when considering only rotation, such as in ArUco OpenCV or ArUco MIP families.

## 5. Conclusions

This paper presents the first study specifically focused on the generation and detection of reflection-aware dictionaries of square markers.

First, we presented a new formulation for the inter-marker distance which considers the possibility of marker reflection. Secondly, a new method for generating reflection-aware dictionaries maximizing the inter-marker distance was proposed. As proved in the experimentation section, our proposal clearly outperforms all other tested dictionaries, doubling the minimum distance in almost all cases and, therefore, doubling the bit-correction capacity. Moreover, part of the proposed method consists of a dictionary optimization that can also be applied to preexisting dictionaries. Applying this method to the public dictionaries yields a significant improvement of the inter-marker distances. The optimization can also be applied considering only rotation and still improves most of the preexisting dictionaries. Furthermore, in conjunction with the dictionary generation process, we proposed an algorithm to identify reflection-aware markers that allows, in addition to identifying the marker, to report whether it has been detected as reflected or not.

Finally, the source code of the proposed algorithms has been made publicly available as Supplementary Material to this paper.

## Data Availability

Not applicable.

## Supplementary Materials

An implementation of the proposed methods based on the OpenCV ArUco module is available at: https://github.com/sergarrido/opencv_contrib/tree/aruco_reflection (accessed on 30 August 2022).
